# Medical Books as Advertisements for Their Authors

**Published:** 1873-11-15

**Authors:** 


MEDICAL EXAMINER.

*A Semi-Monthly Journal of Medical Sciences.*

EDITED BY

N. S. DAVIS, M. D., AND F. H. DAVIS, M. D.

Chicago, Nov. 15th, 1873.

### EDITORIAL.

### MEDICAL BOOKS AS ADVERTISEMENTS FOR THEIR AUTHORS.

"Of the making of books there is no end;" and to the class of medical books is this saying especially applicable. Their numbers are legion—and still they come!—new names pressing onward for rank and fame in the crowded lists of authorship. Pick up the catalogue of any one of the prominent medical book publishing houses and note the contents—books, great and small, thick and thin, quartos, octavos and duodecimos; works on every conceivable branch or special topic appertaining to medicine or surgery; and not one, but a dozen, mayhap, to choose from in each department.

And yet, as compared with general literature, medical publications have but a very limited field for circulation. Book-sellers claim that medical books offer but small profit and very slow sale; indeed, the demand, except for a few standard works, is not sufficient to warrant the keeping them in stock by ordinary dealers. How, then, can all these works be profitable? Directly, they are not so; the large majority of them never sell a sufficient number of copies to pay one-half the first cost of issue. Neither, in many instances, was it expected that they would.

A physician wishes to push a certain line of practice, and obtain name and reputation in that department. Perhaps he has not sufficient influence to command a college or hospital appointment: or he may reside where no such institutions exist. Straightway he goes to work and writes a book—great or small—according to the amount of money that he wishes to invest. In this the literature of his subject is re-hashed and cooked over, and the stolen ideas presented as original, whenever he is not likely to be found out. Above all, however, his own peculiar success in the diagnosis and treatment of this special class of affections is set forth in glowing colors. He then contracts with a publishing firm to issue his work, he paying for the cost a sum sufficient to enable the publisher to realize a fair profit on the printing and making of the book. His publisher then sells the book for him—*if he can*. It is, of course, reviewed and advertised in all the medical journals, and everywhere the author's name appears prominent. The profession throughout the country is thereby informed that Dr. A. or Dr. B. is the author of a work, and, of course, authority on the subject considered. Patients come flocking in for consultation from all directions. The doctor's fortune is made; and still he has done no unprofessional advertising—only invested some five to fifteen hundred dollars in a book that probably did not sell. Nobody but himself and his publisher, however, are aware of that. You did not buy the book, but you saw it noticed and you supposed likely your neighbor did. The books lie on the publisher's shelves, not a dozen copies, perhaps, sold; and yet both author and publisher have cleared a good profit from the investment. Almost any book, no matter how worthless, will be likely to sell a few copies. In the confusion consequent on the multiplicity of books on a given subject, many buyers may fail to distinguish the worthy from the worthless—the wheat from the chaff. The really meritorious books are quite as likely to be overlooked—drowned quite out of sight.

In England they have carried this science of professional advertising a step further. The authors cause their books to be advertised in the secular newspapers; and even have hand-bills posted on the street corners announcing "The new work on *Uterine Tumors*, by Dr. A.;" or "Dr. B's Treatise on *Throat Diseases, etc.*"—with the evident intention, not of pushing the sale of the work, but of calling the attention of the public to the fact that Dr. A., or Dr. B., is an author, and a leading authority on a certain subject.

Lately there has sprung up a very bitter discussion in the English journals regarding this matter, originating in the action of the council of the London College, who have formally condemned the proceeding, and prohibited their members from advertising their books in this way.

It is claimed by some that the established physicians, who constitute the council, desiring to maintain undisputed possession of their respective cocklofts, have kicked away the ladder up which they have climbed. One "Medicus" asserts that to stop advertising his books would "retard his advance in practice ten years, and keep him starving;" and pointedly calls attention to the fact that the works of Mr. Carling, president of the college, have been advertised to such an extent as to make Carling and testis household words in the community, and consequently to establish their author. Another correspondent alludes pleasantly to the circumstance that the London *Lancet*, which has strenuously upheld the council, placards the railway stations with the titles of communications on "*Tumors of the Womb*, "*Rectum*," and other subjects generally supposed not to offer edifying reading for mixed companies of ladies and gentlemen. The defense offered for the president of the council in the matter seems to be that, having sold his works to the publisher he has no control over them. This will undoubtedly be seized upon by others as a convenient mode of circumventing the action of the council.

The contributing to our professional literature, either in the way of independent treatises or of communications to the medical periodicals, is, of course, a legitimate and desirable method of pushing professional reputations, as well as of contributing to the advancement of medical science, always providing that one has something of real value and importance to communicate. It is to the too numerous class of writers who strive only for self-laudation, and to expatiate upon their own extraordinary skill and ability, that we take exception. While direct advertising would at once place them without the ranks of the regular profession, yet they are enabled in this manner to accomplish exactly the same purpose, with probably less expense to themselves, and without in any way violating the code of medical ethics.

The means of correcting this abuse rests with the publishers. Let them be more careful to scrutinize the communications that they receive for their periodicals, and more stringent as to the character of the books upon which their imprint appears.

In general literature, the imprint of a popular publishing house is an almost invariable guarantee for the value and success of a work. If the leading *medical* publishers would be equally careful of their reputation, there would be a much fewer number of books issued, and the profession would have some guarantee that the books published were really worthy of perusal and study.

